# Skin biomechanical and viscoelastic properties measured with MyotonPRO in different areas of human body

**DOI:** 10.1111/srt.13116

**Published:** 2021-11-09

**Authors:** Katarzyna Rosicka, Barbara Mierzejewska‐Krzyżowska, Włodzimierz Mrówczyński

**Affiliations:** ^1^ Department of Biological Sciences Faculty of Physical Culture Poznań University of Physical Education Gorzów Wielkopolski Poland; ^2^ Department of Neurobiology Faculty of Health Sciences Poznań University of Physical Education Poznań Poland

**Keywords:** biomechanical, human, MyotonPRO, skin, viscoelastic

## Abstract

**Background:**

There is still a lack of clinically practical device, which allows to perform rapid and accurate examination of the skin condition. For this reason, suitability of the MyotonPRO for the assessment of skin biomechanical and viscoelastic parameters was evaluated in this study. The aim of the study was to establish the reference values of five parameters measured by MyotonPRO various locations of human skin.

**Materials and methods:**

Oscillation frequency, dynamic stiffness, logarithmic decrement, mechanical stress relaxation and creep were measured at three different skin locations (clavicula, volar forearm and shin), using L‐shape short and medium arm probes in 32 young female volunteers. Mean values of obtained parameters recorded by both probes were compared among three skin locations while reliabilities of measurements were assessed. Additionally, relationships between all recorded parameters were examined

**Results:**

There were no statistically significant differences between the mean values of five measured parameters obtained with both probes in all investigated areas. However, statistically significant differences of mean values of almost all parameters measured among three places examined were found. Despite considerable differences in mean values of obtained parameters, there were visible strong correlations between some studied parameters in all three investigated areas of skin.

**Conclusion:**

It was demonstrated in all locations studied that the higher value of oscillation frequency corresponds to the higher value of dynamic stiffness, moreover such tissue recovers faster to its initial shape, and it was characterized by lower creep values. Such results indicate the existence of identical relationships between the same studied parameters in different areas of skin.

## 1 INTRODUCTION

Devices used for skin evaluation vary by direction of the force/load used for measurement, that is, horizontal, vertical, linear, as well as for measurement of biomechanical properties. Indentometer measures skin compliance/stiffness,^1^, ^2^ CutiScan measures viscoelasticity and anisotropy,^3^, ^4^ Reviscometer describes acoustical shock wave hardness,^5^, ^6^, ^7^, ^8^ Frictiometer defines skin friction,^5^, ^7^, ^9^ Ballistometer evaluates skin elasticity and hardness,^10^, ^11^, ^12^, ^13^ ultrasound elastography uses Young's modulus to describe tissue stiffness,^5^, ^14^, ^15^, ^16^, ^17^, ^18^, ^19^ DermaLab/ DermaLab Combo define elastic properties,^20^, ^21^, ^22^, ^23^, ^24^, ^25^ SkinFibrometer measures skin induration^5^, ^26^, ^27^, ^28^, ^29^ and most commonly used Cutometer measures skin elastic and viscoelastic properties.^5^, ^7^, ^10^, ^26^, ^30^, ^31^, ^32^, ^33^, ^34^

Suction methods are one of the most widely used in basic and in applied skin research.^5^, ^35^ The most commonly used and investigated device ‐ Cutometer is based on suction method, it uses negative pressure to deform the skin mechanically.^5^, ^7^, ^10^, ^26^, ^30^, ^31^, ^32^, ^33^, ^34^ Although Cutometer measures skin elastic and viscoelastic properties, it was stressed that the number of described parameters should be reduced.^5^, ^7^ Moreover choosing proper parameter might be difficult since some descriptors has not been correlated or assigned to physiological functions or morphological components of skin.^5^ Furthermore obtained parameters should be expressed in standard SI units for greater clarity.^5^

CutiScan is also based on suction method and describes viscoelastic properties as well as skin anisotropy.^3^, ^4^, ^36^ However viscoelastic properties are not yet defined with any standard SI units. V1 parameter shows the maximum displacement, V2 – represents the ability to retract to its initial shape, and V3 is a ratio of V2/V1. Moreover this device was not widely tested, and there is still lack of studies conducted with CutiScan.

Ballistometer involves measurements of the skin surface after it has been struck by a known mass with a known force.^10^, ^11^, ^12^, ^13^ The five parameters obtained with this device are correlated with hardness and elasticity of the skin. It was reported that Ballistometer is useful especially while assessing hard skin areas like on forehead.^10^ Although again parameters recorded by this device are not described with standard SI units.

DermaLab Combo is a device that enables ultrasound examination as well as measuring different parameters such as skin pH, viscoelasticity, pigmentation, temperature, hydration and thickness.^20^, ^21^, ^22^, ^23^, ^24^, ^25^ Main biomechanical parameters are based on suction applied to the skin surface and are expressed in standard SI units. DermaLab Combo was used to investigate skin properties in healthy subjects,^20^ to evaluate scars^21^, ^22^, ^23^, ^24^ and to asses effectiveness of treatment in plaque psoriasis.^25^ Study conducted on healthy subjects confirms good to excellent reliability for inter‐rater reliability in most investigated areas.^20^ However, it is not easily accessible, and in order to perform ultrasound examination, it requires appropriate investigator training.

Due to wide range of devices available on market, various technologies are based on as well as differences in parameters measured by each device and/or values obtained, often are not compatible to others devices, results are difficult to compare and interpret their relevance and clinical perception.^5^ Hence, it is necessary to develop a device dedicated for a practical clinical use that will enable the quick and accurate non‐invasive assessment of skin bio‐mechanical properties. Such approach will contribute to considerably more accurate assessment of skin condition and as a result to reliable diagnosis of its state.

MyotonPRO device seems to offer innovative solution for objective palpation. Till now, it was widely tested mainly on muscles, tendons and ligaments.^37^, ^38^, ^39^, ^40^, ^41^, ^42^, ^43^ Although the most commonly used parameters for muscle measurements were stiffness, elasticity and tone, few research contain all five parameters (oscillation frequency, dynamic stiffness, logarithmic decrement, mechanical stress relaxation time and creep) included in MyotonPRO technology.^42^, ^43^

MyotonPRO can also be used to measure the biomechanical and viscoelastic properties of the skin. Previous studies using MyotonPRO were performed only in healthy subjects^44^ and in patients with cutaneous chronic graft‐versus‐host disease (cGVHD) for evaluate skin stiffness.^45^ Furthermore it was used for the assessment of post‐caesarean section scars, and obtained results were compared with unscarred skin.^46^ Moreover, a greater reliability of this device using L‐shape probes for assessment of skin stiffness was demonstrated in our previous report.^47^

Therefore, the main purpose of this study was to establish the reference values (mean, ranges) of all five parameters (oscillation frequency, dynamic stiffness, logarithmic decrement, mechanical stress relaxation time and creep), simultaneously recorded by MyotonPRO in three different areas of human body, which are characterized by different proportion of superficial and deeper tissues (clavicula, volar forearm and shin). The secondary goal was to compare obtained values of all collected parameters, among three locations investigated. Moreover, correlations occurring between all studied parameters were also examined in each investigated place and compared in order to verify if relationship of parameters is constant. We assumed that establishing the reproducibility of the correlations between parameters in different skin locations will provide strong evidence that MyotonPRO can be consider as a reliable device for skin assessment.

## 2 MATERIALS AND METHODS

Study was focused on evaluation of skin biomechanical and viscoelastic properties using MyotonPRO (Myoton AS, Estonia).

### 2.1 Sample size estimation

Sample size was estimated using an a priori power analysis (G ‐ Power software v.3.1.9.4) for repeated measures analysis of variance (ANOVA) using the following parameters (power = 0.95, alpha = 0.05, effect size = 0.60) and revealed that a minimum of 28 participants would be sufficient. Moreover, sample size was estimated according to the European Group on Efficacy Measurement and Evaluation of Cosmetics and other Products guidance for the in vivo assessment of Biomechanical Properties of the Human Skin, that is, minimal number of participants is 30.^5^

### 2.2 Subjects

All subjects initially completed a health screening questionnaire to assess eligibility for the study. As a result, 32 healthy Polish women at the age 19–25 (mean age: 20.6 ± 1.7) and with BMI ranged from 18.3 to 29.3 (mean BMI: 22.5 ± 2.7) were recruited across physiotherapy students (Faculty of Physical Culture in Gorzów Wielkpolski, University of Physical Education in Poznań). Non‐smokers were exclusively selected for the study, without skin diseases, scars, tattoos and wounds on the examined skin areas. Subjects were fully informed about the procedures and potential risks involved in the experiment and provided written consent to participate in the experiment. We decide to include in the study all recruited volunteers who met our participation criteria (32) despite the fact that a sufficient number of people participating in the study were 28. We expected this will get results more representative of the entire population.

### 2.3 Experimental design

Each participant spent 5 min in examination room in order to acclimatize before measurements were taken. Multi scan measurement contains five single measurements (device delivers five short mechanical impulses), and it is automatically presented as the average of this consecutive measurements. Moreover multi scan measurements were repeated five times in all three examined areas in each patient. Average over the five repetitions was used in subsequent analysis. Multi scan measurement for one reference point took about 5 sec, while the total examination time for one probe in each location was less than a minute. During measurements, each subject laid supine in relaxed and comfortable position on the exam table for shin and volar forearm measurements and sat for clavicula measurements. Each examination session was held between 8 and 12 am and was performed by observer who has 2 years’ experience with using MyotonPRO.

Measurements were conducted on three marked sites – clavicular, volar forearm and shin, preferably on dominant upper and lower limb. Clavicula and shin area were analysed due to its relatively thin hypodermis layer. Volar forearm was chosen, because it is one of the most investigated areas in skin research.^31^ The measurement site for clavicula was in the corpus claviculae area, where muscles has no its origin or insertion. The measure spot on the volar forearm was taken in the upper third of forearm. The measurement site for shin was on margo anterior in most palpable area.^47^ Measurement on shin area and volar forearm were performed across Langer's tension line,^48^, ^49^, ^50^, ^51^ while measurement on the clavicula area was collected diagonally to Langer's line. Presented study was performed in accordance with ethical guidelines of Helsinki Declaration and approved by the independent Bioethics Committee of Poznań Medical University, Poland (no. 59/20).

### 2.4 Skin biomechanical and viscoelastic properties

**FIGURE 1:**
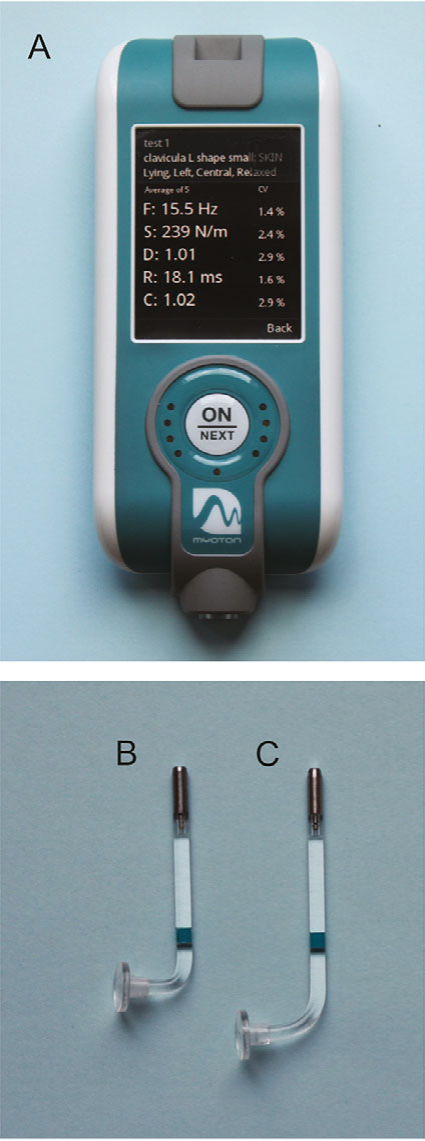
MyotonPRO (A), short arm L‐shape probe (B), medium arm L‐shape probe (C)

The biomechanical and viscoelastic properties of the skin in the above‐mentioned places were examined with two L‐shaped probes, that is, short arm probe (SAP) ‐ length 15 mm and medium arm probe (MAP) ‐ length 20 mm with a disc attachment (diameter 10 mm) designed by Myoton AS and designed specifically for measuring human skin (Figure 1).

Following parameters were noted by both used probes: (1) oscillation frequency (Hz) which represents tissue's intrinsic structural state of tension on cellular level, two biomechanical properties, that is, (2) dynamic stiffness (N/m), which indicates the resistance to an external force which deforms tissue from its initial shape and (3) logarithmic decrement of tissue's natural oscillation, which describes its elasticity and dissipation of mechanical energy when tissue recovers from being deformed, two viscoelastic properties such as (4) mechanical stress relaxation time (ms), which indicates the time needed for tissue to recover to its initial shape after removal of an external force and (5) creep, which indicates a gradual elongation of tissue over time, while it is under constant tensile stress.

The device's probes automatically apply a constant pre‐load force (0.18 N). In standard configuration (setting recommended by Myoton AS), the device delivers a short (15 ms) mechanical impulse of 0.4 N under constant biasing, causing the tissue to respond in the form of damped oscillation. The time interval between each pulse is 0.8 s.

The L‐shape probes deliver measurement impulses horizontally along with skin surface without involvement of deeper subcutaneous layers and causing damped oscillation of the skin surface alone. The device is held parallel to the skin surface while being used. In order to obtain firm contact of the probe and skin, a thin (0,1 mm) double‐sided stickers (10 mm diameter) were used.

### 2.5 Reliability

We found high intraclass correlation coefficients and low coefficients of variation for Oscillation Frequency (SAP inter‐class correlation [ICC]: 0.6–0.96 and CV: 4.66–6.55, MAP ICC: 0.7–0.98 and CV: 3.48–6.17), dynamic stiffness (SAP ICC: 0.72–0.98 and CV: 3.22–6.25; MAP ICC: 0.78–0.97 and CV: 3.32–5.1), logarithmic decrement (SAP ICC: 0.7–0.82 and CV: 6.86–8.56; MAP ICC: 0.78–0.85 and CV: 6.74–7.43), mechanical stress relaxation time (SAP ICC: 0.71–0.98 and CV: 4.45–6.29; MAP ICC: 0.78–0.98 and CV: 4.26–5.93) and creep (SAP ICC: 0.68–0.98 and CV: 4.2–6.12; MAP ICC: 0.77–0.98 and CV: 4.17–5.87) for both probes.

**FIGURE 2:**
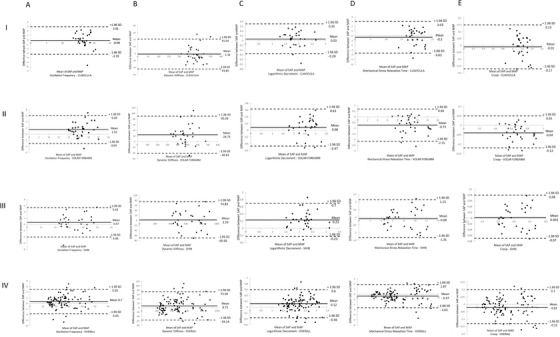
Bland‐Altman plots (including limits of agreement) analysis of the short versus medium arm probe measurements: Columns from left to right: (A) oscillation frequency, (B) dynamic stiffness, (C) logarithmic decrement, (D) mechanical stress relaxation time and (E) creep. Rows from top to the bottom: (I) clavicula, (II) volar forearm, (III) shin and (IV) overall (which represents average values of skin parameters across all measured sites)

The Bland–Altman plots with limit of agreement were used to compare measurements with SAP versus MAP of the same parameter of each site (see Figure 2)

### 2.6 Statistical analysis

Statistical analysis was performed using Statistica 13 software (Statsoft, Poland, Cracow). The normality of the distributions was assessed with the Shapiro–Wilk test. When the same parameters between different locations measured by both L‐shape probes were compared, and the data distribution was normal, a parametric test ‐ repeated measures ANOVA with Tukey's HSD post hoc was used. On the other hand, when the relationships between various recorded parameters were examined ‐ nonparametric analysis (Spearman rank order correlation) was used to evaluate the monotonic relationship/as designed to analyse of the data with not normal distribution. Correlations were classified as trivial (0−0.1), small (0.1−0.3), moderate (0.3−0.5), large (0.5−0.7), very large (0.7−0.9), nearly perfect (>0.9), and perfect (1.0). The differences were set as significant at *p* < 0.05.

## 3 RESULTS

### 3.1 Reference parameters analysed by SAP and MAP probes of MyotonPRO

**FIGURE 3:**
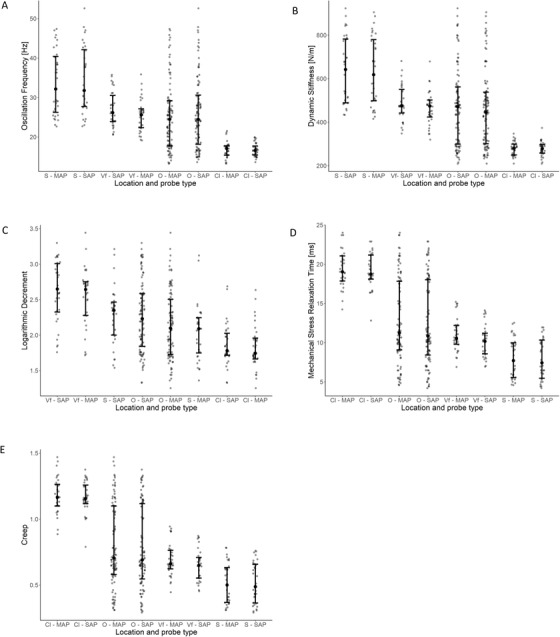
Ranges of all five parameters (i.e., (A) oscillation frequency, (B) dynamic stiffness, (C) logarithmic decrement, (D) mechanical stress relaxation time and (E) creep) measured by short arm L‐shape (SAP), medium arm L‐shape (MAP), probes of MyotonPRO in three studied areas, that is, clavicula (Cl), volar forearm (Vf),shin (S) and overall (O). Centre dot represents medians and lower and upper whiskers shows 25/75 percentiles, respectively

*Oscillation frequency*: The highest mean value of oscillation frequency was obtained in the shin area, slightly lower in volar forearm area, while the lowest in the clavicula area (see Table 1 for details). Differences between the mean values obtained among the three investigated areas were statistically significant (Table 1). However no statistically significant differences were noted between the mean values of oscillation frequency recorded by SAP and MAP probes in each area (*p* > 0.05, Tukey's HSD test, Table 1). These both trends are also visible in Figure 3, presenting differences in the ranges of oscillation frequency recorded by SAP and MAP probes in the three studied places.

**TABLE 1:** The mean values ± SD with interquartile ranges of all five parameters measured by MyotonPRO (i.e., oscillation frequency, dynamic stiffness, logarithmic decrement, mechanical stress relaxation time and creep) at using of L ‐ shape short arm probe (SAP) and L ‐ shape medium arm probe (MAP) in three investigated areas: clavicula (Cl), volar forearm (Vf) and shin (S), number of cases studied *n* = 32 . Overall represents average values of skin parameters across all measured sites

Parameters studied		Oscillation frequency (Hz)		Dynamic stiffness (N/m)		Logarithmic decrement		Mechanical stress relaxation time (ms)		Creep	
Types of L‐shape probes		short arm probe (SAP)	medium arm probe (MAP)	short arm probe (SAP)	medium arm probe (MAP)	short arm probe (SAP)	medium arm probe (MAP)	short arm probe (SAP)	medium arm probe (MAP)	short arm probe (SAP)	medium arm probe (MAP)
	Clavicula(Cl)	16.7 ± 1.7 (15.3; 17.9) ^†^	16.8 ± 2.1 (15.4; 17.8) ^†^	277.1 ± 34 (257.5; 297.8) ^†^	278.3 ± 32.2 (249.2; 300.1) ^†^	1.9 ± 0.3 (1.7; 2.0) ^†^ ^‡^	1.8 ± 0.3 (1.7; 2.0) ^†^ ^§^	19.1 ± 2.2 (18.1; 21.2) ^†^	19.4 ± 2.4 (17.9; 21.1) ^†^	1.2 ± 0.1 (1.1; 1.3) ^†^	1.2 ± 0.1 (1.1; 1.3) ^†^
Areas	Volar forearm (Vf)	27.1 ± 4.5 (23.8; 30.7) ^†^	25.5 ± 4.2 (22.3; 27.3) ^†^	489.9 ± 81.0 (440.8; 554.0) ^†^	465.1 ± 78 (420.4; 502.5) ^†^	2.6 ± 0.4 (2.3; 3.0) ^†^	2.5 ± 0.4 (2.3; 2.8) ^†^ ^¶^	10.3 ± 2.0 (8.6; 11.3) ^†^	11 ± 2.2 (9.8; 12.2) ^†^	0.7 ± 0.1 (0.6; 0.7) ^†^	0.7 ± 0.1 (0.6; 0.8) ^†^
	Shin (S)	34.4 ± 8.6 (27.7; 42.3) ^†^	33.7 ± 8.2 (26.0; 40.6) ^†^	637.5 ± 155.2 (488.7; 784.3) ^†^	634.9 ± 158.1 (497.9; 785.5) ^†^	2.3 ± 0.4 (2.0; 2.5) ^†^ ^¶^	2.1 ± 0.4 (1.7; 2.2) ^†^ ^‡^, ^§^	7.9 ± 2.6 (5.5; 10.3) ^†^	8.0 ± 2.6 (5.5; 10.0) ^†^	0.5 ± 0.2 (0.4; 0.7) ^†^	0.5 ± 0.2 (0.4; 0.6) ^†^
	Overall	26.1 ± 9.2 (17.9; 30.8) ^†^	25.4 ± 8.8 (17.8; 29.3) ^†^	468.2 ± 180.2 (297.8; 564.0) ^†^	459.4 ± 178.6 (300.1; 539.7) ^†^	2.3 ± 0.5 (1.8; 2.6) ^†^	2.1 ± 0.5 (1.7; 2.5) ^†^	12.4 ± 5.3 (8.3; 18.1) ^†^	12.8 ± 5.4 (9.0; 17.9) ^†^	0.8 ± 0.3 (0.5; 1.1) ^†^	0.8 ± 0.3 (0.6; 1.1) ^†^

^†^ No Significant difference between SAP and MAP (*p* > 0,05, Tukey's HSD).

^‡^ No Significant difference between clavicula SAP and shin MAP (*p* > 0,05, Tukey's HSD).

^§^ No Significant difference between clavicula MAP and shin MAP (*p* > 0,05, Tukey's HSD).

^¶^ No Significant difference between volar forearm MAP and shin SAP (*p* > 0,05, Tukey's HSD).

*Dynamic stiffness*: As to the oscillation frequency, the highest mean value of skin stiffness occurs in the shin area, lower in volar forearm area and the lowest in the clavicula area (Table 1). While comparing obtained values of dynamic stiffness in the three examined locations, statistically significant differences were found between them (Table 1). Mean values of dynamic stiffness recorded with both probes were almost the exact the same in each investigated area, therefore no statistical significant differences between them (see Table 1). These trends are illustrated in Figure 3 presenting the ranges of dynamic stiffness values recorded by SAP and MAP probes in all studied sites.

*Logarithmic decrement*: In contrast to both previously described parameters, the highest mean value of logarithmic decrement was observed in the volar forearm area, lower in the shin area and the lowest in the clavicula area (see Table 1). Both mean values (Table 1) and ranges of logarithmic decrement (see Figure 3) varied much less between the studied areas in comparison to previously analysed parameters. Moreover, statistically significant differences were not found between all of the compared areas (see Table 1 for details). In each of the three studied locations, there was no significant difference between the logarithmic decrement obtained with SAP and MAP (Table 1). The ranges of this parameter value recorded by both probes were also closely comparable in each one place studied (Figure 3).

*Mechanical stress relaxation time*: The longest time needed for the skin to recover to its initial shape was noted in the clavicula area, while much shorter in the volar forearm and shin areas (Table 1). However, the mean values of this parameter were significantly different among these three spots (Table 1). On the other hand, similarly to the remaining parameters, there were no significant differences between the mechanical stress relaxation time recorded by MAP and SAP probes in all researched areas (Table 1). The differences of mechanical stress relaxation time ranges recorded by SAP and MAP within each site are shown in Figure 3.

*Creep*: The lowest mean value of creep was recorded in the shin area, slightly higher in volar forearm area, and the highest in the clavicula area (Table 1). Thus, differences between creep values in the evaluated areas were determined as statistically significant (Table 1). Again, the mean values as well as the ranges of creep obtained with both probes did not differ significantly in any one studied place (Table 1, Figure 3).

### 3.2 The relationship between examined parameters

The Spearman's rank correlation coefficient was used to examine the relationships between all parameters recorded by SAP and MAP probes of MyotonPRO in all investigated areas. The data are presented in Table 2.

**TABLE 2:** Spearman rank order correlations used to examine the relationships between skin biomechanical parameters, that is, F‐ oscillation frequency (Hz), S‐ dynamic stiffness (N/m), D‐ logarithmic decrement, R‐ mechanical stress relaxation time (ms), C‐ creep. Overall represents average values of skin parameters across all measured sites

Areas	**Short arm L‐shape probe (SAP)**						**Medium arm L‐shape probe (MAP)**				
		F	S	D	R	C	F	S	D	R	C
***Clavicula (Cl)***	F		0.897	0.163	−0.790	−0.834		0.840	0.389	−0.856	−0.849
	S	0.897		0.230	−0.778	−0.866	0.840		0.381	−0.939	−0.911
	D	0.163	0.230		0.151	0.176	0.389	0.381		−0.204	−0.192
	R	−0.790	−0.778	0.151		0.795	−0.856	−0.939	−0.204		0.990
	C	−0.834	−0.866	0.176	0.795		−0.849	−0.911	−0.192	0.990	
***Volar forearm (Vf)***	F		0.798	0.569	−0.834	−0.840		0.789	0.500	−0.866	−0.878
	S	0.798		0.242	−0.992	−0.986	0.789		0.093	−0.978	−0.970
	D	0.569	0.242		−0.310	−0.335	0.500	0.093		−0.227	−0.250
	R	−0.834	−0.992	−0.310		0.996	−0.866	−0.978	−0.227		0.997
	C	−0.840	−0.986	−0.335	0.996		−0.878	−0.970	−0.250	0.997	
***Shin (S)***	F		0.940	−0.118	−0.960	−0.953		0.956	−0.535	−0.968	−0.965
	S	0.940		−0.248	−0.987	−0.988	0.956		−0.596	−0.987	−0.987
	D	−0.118	−0.248		0.219	0.232	−0.535	−0.596		0.600	0.605
	R	−0.960	−0.987	0.219		1.00	−0.968	−0.987	0.600		1.00
	C	−0.953	−0.988	0.232	1.00		−0.965	−0.987	0.605	1.00	
**Overall**	F		0.969	0.503	−0.965	−0.965		0.968	0.306	−0.975	−0.975
	S	0.969		0.432	−0.987	−0.989	0.968		0.244	−0.995	−0.993
	D	0.503	0.432		−0.424	−0.421	0.306	0.244		−0.251	−0.250
	R	−0.965	−0.987	−0.424		0.992	−0.975	−0.995	−0.251		1.0
	C	−0.965	−0.989	−0.421	0.992		−0.975	−0.993	−0.250	1.0	

*Note*: Correlations were classified as trivial (0−0.1), small (0.1−0.3), moderate (0.3−0.5), large (0.5−0.7), very large (0.7−0.9), nearly perfect (>0.9) and perfect (1.0). Minus (−) represents a negative correlation. All boxes without any shading have two‐tailed *p*‐value >0.05.

## 4 DISCUSSION

Recently, some studies have been conducted to evaluate the biomechanical and viscoelastic properties of human skin^44^, ^45^, ^47^ and post Caesarean section scars^46^ using MyotonPRO. It was previously reported that inter‐observer and intra‐observer ICC values showed great or excellent reliability of the MyotonPRO for stiffness measurement^44^ as well as all other parameters.^46^ Our earlier research has shown that MyotonPRO equipped with L‐shape probes is perfectly suited for measuring skin stiffness in humans.^47^ Majority studies on biomechanical and/or viscoelastic properties of human skin performed with the MyotonPRO were focused on dynamic stiffness of healthy subject,^44^, ^45^, ^47^ while other parameters were not analysed or focused on specific area, only.^46^ This approach is due to the fact that stiffness parameter in the literature is most referred and analysed in human skin studies as it can be very useful for scar evaluation^18^, ^27^, ^46^ and for assessment of certain diseases causing stiffening such as sclerosis in cutaneous cGVHD,^45^ scleroderma^52^ and systematic sclerosis.^16^, ^17^, ^53^

Our result indicated on significant differences in values (means, ranges) of oscillation frequency and dynamic stiffness in the three examined locations. Values of skin oscillation frequency in three location studied by us were not reported previously, while values of skin stiffness obtained by L‐shape MyotonPRO probes in our study for Clavicula (which can be referred to Upper Arm), volar forearm and shin areas seem to be very similar to these noted by MyotonPRO in studies by Dellalana et al.,^44^ Chen et al.^45^ in healthy controls groups and to our previous study carried out on smaller group of patients.^47^

Highest values of both parameters were noted in the shin area, lower in volar forearm area, while the lowest in the clavicula area. Oscillation frequency represents the internal structural tension of the tissue, while dynamic stiffness indicates the resistance to an external force that deforms the tissue from its initial shape. Higher values of these both parameters mean the higher intrinsic tension within the tissue as well greater stiffness of tissue.^43^ Such an order in magnitude of the collected values in these three studied sites could be related to the need to compensate for the increased hydrostatic pressure in lower limbs (calf) comparing to upper extremities (forearm). Such a role has been previously reported in relation to the skin stiffness.^54^ Volar forearm area is less exposed to sunlight therefore the impact of photo‐aging process is lower^6^ and as a consequence is not as thick as other areas of the body.^10^ In females skin thickness on volar forearm is approximately 1.12 mm in contrast to shin area, that is, 1.34 mm.^55^ It was previously reported that skin's biomechanical properties in white Northern Europeans varies among body sites depending on sun exposure.^56^ Furthermore, there is a difference in skin pH between volar forearm ‐ 5.4–5.9 and shin 4.8–5.5.^57^ All above mentioned differences in biological and physical features might contribute to differences in biomechanical properties among different body sites.

It should be taken into consideration that method of performing measurements (direction of the impulse specifically) might also influence on the values of parameters recorded by MyotonPRO. Higher values of oscillation frequency and dynamic stiffness were obtained across comparing to those collected diagonally to Langer's line. Therefore the knowledge of tension lines in skin is essential while planning experimental procedures, since skin is an anisotropic tissue, and consequently it behaves differently depending on direction of applied force.^54^, ^58^

Logarithmic decrement of a tissue's natural oscillation indicates its elasticity and the dissipation of mechanical energy when tissue recovers from being deformed. Elasticity characterises the tissues ability to return to its initial shape when the external force is removed. It should be mentioned that logarithmic decrement is inversely proportional to elasticity, which means the higher the value of logarithmic decrement, the lower the elasticity.

Our result suggest that values of this parameter (means and ranges) recorded by both L‐shape probes were comparable to each other in all three studied locations. The absence of differences of the skin elasticity in three different places studied can be explained by the homogeneity of the age of studied group (women at the age 19–25). It was already reported that elasticity of skin is age‐related parameter,^6^, ^13^, ^31^, ^32^, ^34^ which can be more diverse while comparing skin of younger and older groups participants.

Mechanical stress relaxation time indicates the displacement recovery time when external force of deformation is removed. On the other hand, creep that is indicated by ratio of deformation and recovery time represents total behaviour of skin, that is, viscoelastic properties of skin. During creep test, when skin is submitted to sudden and sustained strain, following phases are found: immediate deformation, purely elastic nature (phase I), viscoelastic deformation (phase II) and late deformation or viscous (phase III).^54^, ^59^ Both parameters are related to each other.

In our study, the lowest values of creep and mechanical stress relaxation time were recorded by both L‐shape probes in shin area, slightly higher in volar forearm area and the highest in clavicula area. Therefore, the faster tissue recovers to its initial shape (smaller values of mechanical stress relaxation time), the smaller was creep value. It was reported on muscles that the smaller values of creep parameter characterises healthier and younger tissues^43^; hence it is possible that such regularity is also characteristic for the skin.

Reference values of all tested parameters were obtained with the use of both L‐type probes, which are intended for testing skin properties, only. For this reason, the results presented above cannot be compared to those obtained with a typical MyotonPRO application using a standard probe with or without disk attachment as well as in longitudinal measurement mode. These limitations should be resolved in future research.

Very large or nearly perfect positive correlations were observed between dynamic stiffness and oscillation frequency in all three studied places. On the one hand, these results lead to the more general conclusion that the dynamic stiffness of the skin has the tendency of increasing along with increasing values of tension within skin. On the other hand, results show that such relationship between studied parameters is permanently present in each location studied and with both probes.

Despite the fact that our study demonstrated large and/or moderate correlations between logarithmic decrement and other parameters in some areas (see Table 2 for details), there was no repeatability with those correlations. It indicates that logarithmic decrement and as a consequence skin elasticity vary less among different areas of the skin especially in people of the same sex within similar age range.

Moreover, very large and perfect positive correlations were noted between mechanical creep and mechanical stress relaxation time in all investigated areas and with both probes.

To sum up, collected data suggest that the higher intrinsic tension of the skin, the higher dynamic stiffness, moreover such tissue recovers faster to its initial shape, and it is characterized by smaller creep value. The above‐mentioned correlations between biomechanical and viscoelastic properties of the skin of participants were almost identical in all three examined locations.

### 4.1 Limitations and future directions

We hope that our research is the first step to evaluating biomechanical and viscoelastic properties of human skin and revealing correlations occurring between those parameters. We examined above mentioned parameters of the clavicula area, volar forearm and shin area skin in young healthy females. It should be stressed that elasticity (logarithmic decrement) is age‐related feature, ^6^, ^13^, ^31^, ^32^, ^34^ and our results may have limited generalisability to older or clinical populations. Furthermore, similar study should be conducted on a representative group of males. Moreover, while designing research protocol, some aspects of skin mechanical behaviour should be taken into consideration, that is, skin anisotropy, since some parameters seem to depend strongly on force direction. Also, our research examined only limited areas of skin, and future research should focus on different areas of the human body to provide improved understanding of skin biomechanical and viscoelastic properties. In addition, the data obtained with the L‐probes should also be compared with these recorded by the standard MyotonPRO probe as well as with these obtained with various devices such as the Cutometer at each patient measurement site. Such studies would be necessary to find relationships between measurements made on the skin with different tips and devices and would facilitate the comparison of results obtained with different measurement devices.

## 5 CONCLUSION

This study demonstrated that MyotonPRO equipped with any of L‐shape probes, that is, short arm or MAP can be considered as a skin biomechanical/viscoelastic properties assessment device. This conclusion is due to the fact that MyotonPRO in each tested location was able to record values of five different parameters of the skin, which together allows to describe its basic biomechanical and viscoelastic properties. Moreover, despite the differences in obtained values of parameters, correlations occurring between them were almost identical among all there examined locations

## CONFLICT OF INTEREST

The authors have no relationship that could lead to conflict of interest. None of the authors have any competing interest.
